# Endothelial Cell HIF-1α and HIF-2α Differentially Regulate Metastatic Success

**DOI:** 10.1016/j.ccr.2011.11.017

**Published:** 2012-01-17

**Authors:** Cristina Branco-Price, Na Zhang, Moritz Schnelle, Colin Evans, Dörthe M. Katschinski, Debbie Liao, Lesley Ellies, Randall S. Johnson

**Affiliations:** 1 Department of Physiology, Development and Neuroscience, University of Cambridge, CB2 3EG Cambridge, UK; 2 Department of Biology, University of California, San Diego, La Jolla, CA 92093, USA; 3 Department of Pathology, University of California, San Diego, La Jolla, CA 92093, USA; 4 Department of Cardiovascular Physiology, Universitätsmedizin Göttingen, Georg-August University Göttingen, D-37073 Göttingen, Germany

## Abstract

The hypoxia inducible transcription factors (HIFs) control many mediators of vascular response, including both angiogenic factors and small molecules such as nitric oxide (NO). In studying how endothelial HIF response itself affects metastasis, we found that loss of HIF-1α in endothelial cells reduces NO synthesis, retards tumor cell migration through endothelial layers, and restricts tumor cell metastasis, and that loss of HIF-2α has in each case the opposite effect. This results from differential regulation of NO homeostasis that in turn regulates vascular endothelial growth factor expression in an NO-dependent feedback loop. These opposing roles for the two HIF factors indicate that both they and endothelial cells regulate metastasis as malignancy progresses.

## Highlights

► Endothelial cell hypoxic response is a critical determinant of metastatic success ► HIF isoforms differentially regulate NO homeostasis in endothelium ► iNOS is essential for hypoxic NO production in endothelial cells ► HIF isoforms in endothelium differentially promote and hinder metastasis

## Significance

**There is a central role for endothelial cells in the process of metastasis: they represent a critical barrier to the passage of tumor cells in their migration toward other organs. Vascular damage, clotting, and ischemia are correlated with tumor metastasis, and all involve hypoxic insult to the endothelium. This study demonstrates that the endothelial cell HIF response is complex, and can act to both promote and retard metastasis, dependent on the HIF isoform expressed and the overall regulation of endothelial nitric oxide production via hypoxic response.**

## Introduction

There is a link between the metastatic process and oxygen deprivation (Brizel et al., 1996; Rofstad et al., 2010; Voss et al., 2011). Hypoxia itself triggers the induction of the hypoxia inducible (HIF) transcription factors; these in turn are linked to changes in the capacity of tumor cells to migrate, undergo epithelial to mesenchymal transition, and to a number of other processes intrinsic to metastasis (Chen et al., 2010; Haase, 2009; Liao et al., 2007; Yang et al., 2008). Hypoxic response via HIF activation also includes expression of factors such as vascular endothelial growth factor (*VEGF*), and inducible nitric oxide synthase (*iNOS*) that are known to facilitate both angiogenesis and tumor cell access to the circulatory system (Ambs et al., 1998; Claffey et al., 1996; Shweiki et al., 1992; Ziche and Morbidelli, 2009).

The role of the tumor cell in metastasis has been widely examined and discussed (Chambers et al., 2002; Gupta and Massagué, 2006). Significant evidence exists to indicate that migration of tumor cells during hematogenous metastasis is accelerated by a HIF-driven response (Liao et al., 2007; Lu et al., 2010; Zhong et al., 1999). This response in the metastatic cell may be driven in part by alterations in tumor oxygenation, which also impacts the tumor endothelium, and it follows that the hypoxic endothelial cell (EC), because of its location, could act as a gatekeeper to metastatic cell intravasation.

As intravasatory processes are related to the hypoxia found throughout many solid tumors, hypoxia is also tied to the extravasation process, through the arrest of migrating tumor cells in capillary beds. The resultant thrombus formation can also result in local hypoxia and ischemia, and reemergence of a growing tumor through a second endothelial barrier. Evidence to support this model includes the clear relationship between thrombus formation and metastatic success (Amirkhosravi et al., 2010; Cliffton and Grossi, 1974; Ostrowski et al., 1986; Palumbo et al., 2005; Saito et al., 1980).

Hypoxia has potent effects on regulation of nitric oxide (NO) (Melillo et al., 1995; Olson and van der Vliet, 2011; Takeda et al., 2010). NO can affect the overall vascular tension of tumor vessels, and also has numerous other localized effects on vascular function (Gunnett et al., 2005; Ignarro et al., 1987; Palmer et al., 1987). Previous work has demonstrated that NO synthesis in EC under normoxia is controlled primarily by endothelial NO synthase (eNOS) (Fukumura et al., 2001). However, hypoxic response induces NO chiefly via iNOS. Intriguingly, some reports have indicated that iNOS itself can positively regulate *VEGF* expression during tumorigenesis (Van der Wall and Palmer, 2006; Wang et al., 2001).

We have recently shown that in inflammatory cells, NO homeostasis is modulated by differential expression of the HIF isoforms HIF-1 and HIF-2 (Takeda et al., 2010) via regulation of two opposing uses of l-arginine: HIF-1α-induced expression of *iNOS*, and HIF-2α induced expression of arginase 1 (*ARG1*), which can remove l-arginine from NO synthetic pathways and thus reduce NO levels. The primary aim of this study was to investigate the effect of EC-specific deletion of HIF-1α or HIF-2α on NO homeostasis and metastatic progression.

## Results

### Loss of HIF-1α in Endothelial Cells Reduces Metastatic Rate

**Figure 1:**
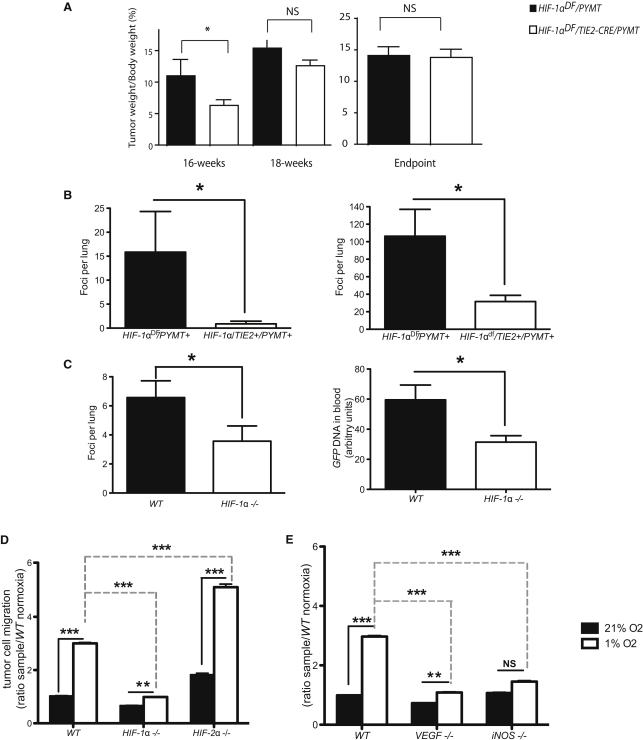
*HIF-1*α Deletion in Endothelial Cells Leads to Reduction in Metastatic Incidence in Transgenic and Xenograft Models (A) Effect of Tie2-driven deletion of HIF-1α on primary tumor growth in the spontaneous breast tumor model PyMT (*HIF-1*α*^df^*/*Tie2Cre*/*PyMT*) during different stages during tumor development, at 16 and 18 weeks, and at endpoint (when one tumor reaches diameter of 1 cm). (B) Effect of Tie2-driven deletion of *HIF-1*α on spontaneous breast tumor model *PyMT* (*HIF-1*α*^df^/Tie2Cre/PyMT*) tumor metastases at 16 wks of age (left panel), and at endpoint (when one tumor reaches diameter of 1 cm, right panel). (C) LLC^GFP^ cells were implanted in cleared mammary pads of endothelial cell-specific deletion of *HIF-1*α (HIF-1α null) obtained by regenerating *WT* bone marrow into *HIF-1*α*^df^/Tie2cre+* mice. Effect of endothelial *HIF-1*α deletion on frequency of lung metastasis 3 weeks after tumor cell implantation (left) and on blood genomic *GFP* levels, a measure of relative abundance of circulating tumor cells (right); Data in (A–C) are average ± SEM. (D and E) Boyden assay for tumor cell migration during 9h periods of normoxia (21% O_2_) or hypoxia (1% O_2_) through endothelial monolayers of *WT*, *HIF-1*α *null*, and *HIF-2*α *null* cells (D) and *VEGF null* and *iNOS null* cells (E). Data in (D) and (E) are average ± SD of ratio of cells migrated through endothelium divided by the mean number of migrated cells through *WT* endothelium during normoxic conditions. An asterisk is placed above differences with a significance exceeding p < 0.05; ^∗∗^p < 0.01; ^∗∗∗^p < 0.001. NS indicates differences that do not reach a p < 0.05 level of significance.

To examine HIF function and endothelial hypoxic response in metastasis, metastatic success was assayed in murine progressive transgenic tumorigenesis, utilizing the *MMTV-PyMT* model of mammary cancer (Lin et al., 2003). These mice, maintained on a C57/Bl6 inbred background were crossed to mice carrying a conditional deletion of *HIF-1*α (Ryan et al., 2000) and the *TIE2CRE* transgenic strain (Kisanuki et al., 2001). The expression of the cre recombinase driven by the Tie2 promoter causes deletion in conditionally targeted endothelial cells and bone marrow-derived cells (Constien et al., 2001). In these first experiments, and in keeping with previously published results (Tang et al., 2004), overall effects on primary tumor growth were only moderate (Figure 1A); however, metastatic success was strongly reduced. At 16 weeks of age, when lung metastatic foci first become evident in this model, they are virtually absent in the *Tie2Cre+ HIF-1*α*^df^* mice (Figure 1B, left). This reduction in metastasis is also evident at the endpoint of the model, where overall primary tumor burdens are similar in wild-type and mutant animals (Figure 1A); but where there is still an ∼75% reduction in numbers of metastatic pulmonary foci (Figure 1B, right). At the terminal stage in this model, there was no significant difference in vascular density in the mutant mouse tumors relative to wild-type animals vascular density (data not shown).

Bone marrow transplantations of wild-type (*WT*) marrow were then introduced into control and mutant mice; this is necessary to circumvent bone marrow expression of the cre recombinase in *Tie2+cre* mice, and results in mice with a deletion solely in endothelial cells (Tang et al., 2004). When GFP-tagged Lewis lung carcinoma cells (LLC) were introduced into mammary fat pads and allowed to grow for 3 weeks, the size of the primary tumor in the different hosts was again identical as were vessel densities (data not shown), but the number of pulmonary foci was significantly reduced (Figure 1C, left). Further analysis of the number of circulating GFP-tagged tumor cells in the bloodstream at the time of sacrifice indicated tumor cells in the circulation were reduced by ∼50% in tumor-bearing *HIF-1*α endothelial cell deletion mutants (Figure 1C, right). This indicates that HIF-1α in the endothelium plays a significant role in determining the number of intravasating tumor cells in this model.

### Migration of Tumor Cells through Primary Endothelial Cell Layers Is Differentially Controlled by HIF-1α and HIF-2α

To better understand the mechanisms underlying HIF control of tumor cell migration in vivo, a tissue culture model of the movement of tumor cells through an endothelial layer was utilized. Primary murine lung endothelial cells were harvested from conditionally targeted mice and treated with cre recombinase-expressing or control adenovirus; they were then assayed for deletion efficacy, and subsequently cultured on an 8 μm filter insert. Labeled tumor cells were then introduced to the control or nullizygous endothelial cell layers and exposed to normoxia or hypoxia (1% O_2_) for 9 hr, after which tumor cells that migrated through the endothelial cell layer were counted. As can be seen in Figure 1D, hypoxia acts to accelerate the migration of tumor cells through wild-type endothelial cells in this system. However, this increased migration is very significantly reduced when endothelial cells lack HIF-1α (Figure 1D). Conversely, endothelial cells lacking HIF-2α (Gruber et al., 2007) exhibit the opposite phenotype: there, migration of tumor cells through the mutant endothelial layer is accelerated relative to wild-type cells under both normoxic and hypoxic conditions.

### Deletion of Both iNOS and VEGF Inhibits Tumor Cell Migration through Endothelial Cell Layers during Hypoxia

Two HIF target genes known to produce mediators of endothelial cell permeability are the angiogenic factor vascular endothelial growth factor-A, or *VEGF-A*, and the inducible NO synthase, or *iNOS* (or *NOS2*). To determine whether loss of either gene in endothelial cells affects the migration of tumor cells in this assay, primary lung *iNOS* null endothelial cells from global deletion animals, and VEGF-A conditional null (Gerber et al., 1999) primary lung endothelial cells (the latter treated ex vivo with cre recombinase-expressing virus, as above) were isolated. Loss of both endothelial *VEGF-A* and *iNOS* restricts the hypoxia-induced migration of tumor cells through the endothelial monolayer (Figure 1E).

### Differential Regulation of VEGF Is Influenced by iNOS Expression during Hypoxia

**Figure 2:**
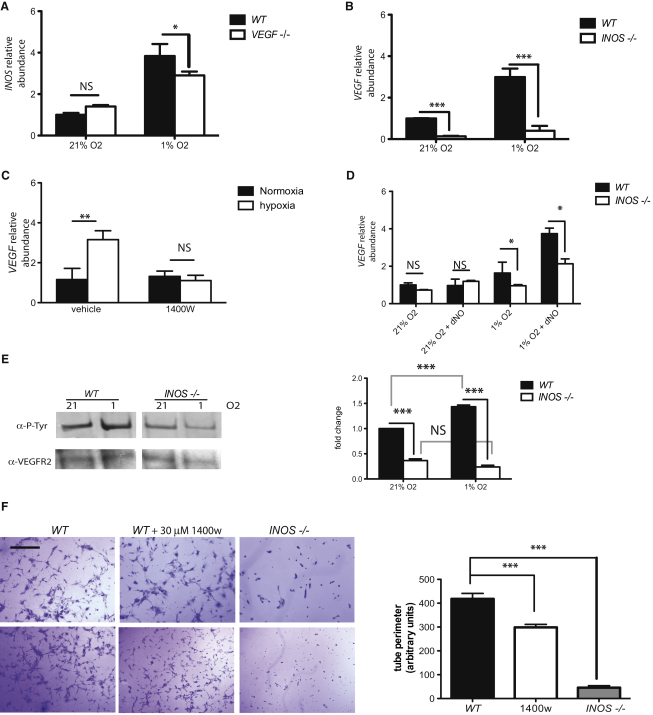
Deletion and Specific Inhibition of iNOS Result in Decreased VEGF Expression and Downstream Activity (A–C) Quantification of relative abundance of *iNOS* mRNA in *VEGF null* endothelial cells (A) and *VEGF* mRNA abundance in *iNOS KO* cells (B) or *WT* cells treated with 15 μM of specific iNOS inhibitor 1400W (right panel) by quantitative RT-PCR (C). (D) *VEGF* mRNA levels in *WT* and *iNOS null* endothelial cells upon addition of 5 mM NO donor DETA NONOate (dNO). (E) Representative western blot of immunoprecipitated VEGFR2 (Flk1) probed for total Flk1 and P-tyrosine to detect activated form (left) and signal quantification of multiple blots (n = 3) by phosphor imaging (right). (F) Tube formation assay in collagen I matrix, representative pictures of *WT*, *WT* + 30 μM 1400W, and *iNOS null* are shown (left) (scale bar represents 100 μm) and quantification of length of detected networks (right) was performed using ImageJ. Data is average ± SEM of lengths obtained from at least three pictures of at least three wells. Experiment shown is representative and was repeated three times with cells no older than passage 2. In this figure, data shown is average ± SEM, An asterisk is placed above differences with a significance exceeding p < 0.05; ^∗∗^p < 0.01; ^∗∗∗^p < 0.001.

As shown above, loss of either *iNOS* or *VEGF* in endothelial cells inhibits transmigration of tumor cells in a cell culture assay. To determine whether iNOS or VEGF is up- or downstream during hypoxic signaling in endothelial cells, *iNOS* mRNA expression was assayed in primary *VEGF null* endothelial cells during hypoxia (Figure 2A); and similarly, *VEGF-A* expression was assayed in *iNOS null* endothelial cells under the same conditions (Figure 2B). Loss of *VEGF* in endothelial cells has no significant effect on expression of *iNOS* in normoxia, but did affect hypoxic induction of iNOS (Figure 2A). However, loss of *iNOS* in endothelial cells suppresses *VEGF* expression in both normoxia and hypoxia (Figure 2B), as does specific inhibition of iNOS with 1400W in WT EC (Figure 2C). To determine whether this effect on *VEGF* expression in endothelial cells is NO-dependent, an NO donor (DETANONOate) was added to cultures to a concentration of 5 mM; this addition resulted in restored VEGF expression under hypoxia in *iNOS null* cells (Figure 2D). This demonstrates that the hypoxic induction of *VEGF* in endothelial cells is regulated by iNOS-mediated production of NO.

### Endothelial Cell Expression of VEGF Is Suppressed by an iNOS-Specific Inhibitor in a Tissue-Specific Fashion

The inhibition of hypoxic induction of autocrine VEGF-A would be expected to reduce hypoxic activation of the Flk-1 (VEGFR2) receptor by phosphorylation, and this is indeed seen in *iNOS null* primary endothelial cells (Figure 2E, left, and phosphor imaging quantification, right). Immunoprecipitation of VEGFR2 from primary lung endothelial cells followed by detection of phosphotyrosine demonstrates that the hypoxic activation of VEGFR2 is significantly reduced in cells lacking iNOS.

VEGFR2 signaling is critical for the induction of tube formation in cultured endothelium (Zhang et al., 2010). To determine whether endothelial cell *iNOS* expression also affects this VEGF-A-dependent phenomenon, cultured EC were placed on collagen matrices, and as shown in Figure 2F, loss of EC *iNOS* reduced tube formation ∼90%. Inhibition of iNOS protein activity by the iNOS-specific inhibitor 1400W (30 μM) also reduced tube formation significantly. These results indicate that iNOS is a critical determinant of function in endothelial cells in isolation.

### Gene Expression Analysis of Hypoxic Endothelial Cells

**Figure 3:**
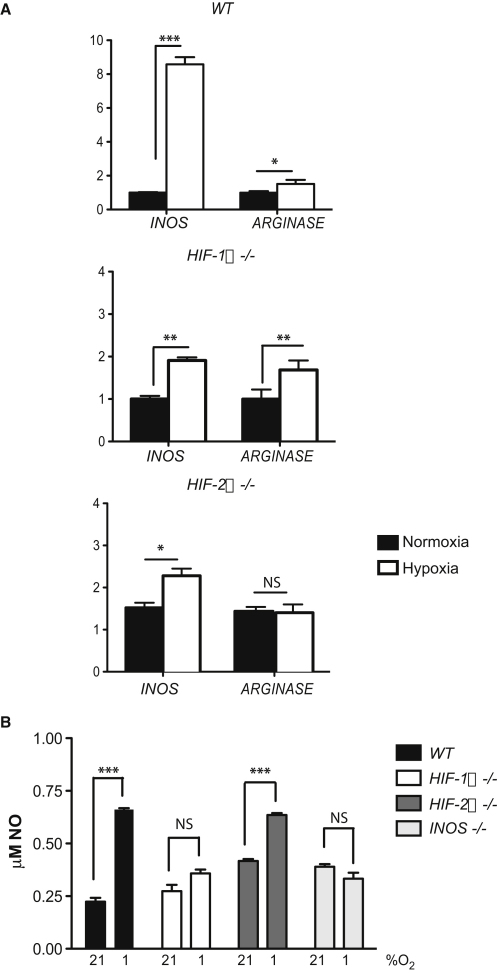
Endothelial Cells Generate NO through iNOS Activation during Hypoxia (A) Relative abundance of *iNOS* and *ARG1* transcripts in *WT* (left), *HIF-1*α *null* (center), and *HIF-2*α *null* (right) endothelial cells exposed to 21% O_2_ (normoxia) or 1% O_2_ (hypoxia) for 9 hr. Data is average ± SEM of each sample; mRNA levels are compared to those observed in WT normoxia (that was defined as 1.0-fold). (B) NO levels in endothelial cell conditioned medium from cells treated for 48 hr at 21% or 0.5% O_2_; data presented are average ± SEM. An asterisk is placed above differences with a significance exceeding p < 0.05; ^∗∗^p < 0.01; ^∗∗∗^p < 0.001.

It was recently shown that loss of *HIF-1*α and *HIF-2*α in macrophages has differential effects on the regulation of NO production; loss of *HIF-1*α resulting in decreased *iNOS* expression and decreased NO levels, and loss of *HIF-2*α in decreased *ARG1* expression and increased NO availability (Takeda et al., 2010). Examination of normoxic expression of *iNOS* and *ARG1* in *WT*, *HIF-1*α, and *HIF-2*α *null* endothelial cells reveals that the two HIF-α factors also regulate *iNOS* and *ARG1* differentially in these cells. Loss of either *HIF-1*α or *HIF-2*α reduced the expression of both *iNOS* and *VEGF* during hypoxia (Figure 3A); however, only the loss of HIF-2α significantly reduced the expression of Arginase1.

### Hypoxic Induction of NO in Endothelial Cells Is HIF-1α and iNOS Dependent

A number of studies have determined that under normoxic circumstances, the primary factor in normoxic endothelial NO production is the endothelial NO synthase (*eNOS*) (Fukumura et al., 2001); however, *eNOS* is neither a HIF target nor a hypoxically responsive gene. To determine the role of iNOS in endothelial cell hypoxia-induced NO production, assays for NO metabolites were carried out on conditioned medium from normoxic or hypoxic endothelial cells. There is a significant increase in NO metabolites in hypoxically conditioned media, and this is dependent on HIF-1α (Figure 3B). HIF-2α loss causes an increase in NO metabolites under normoxic conditions when compared to wild-type cells.

### Inhibition of VEGF Expression by Suppression of iNOS Is Specific to Endothelial Cells

**Figure 4:**
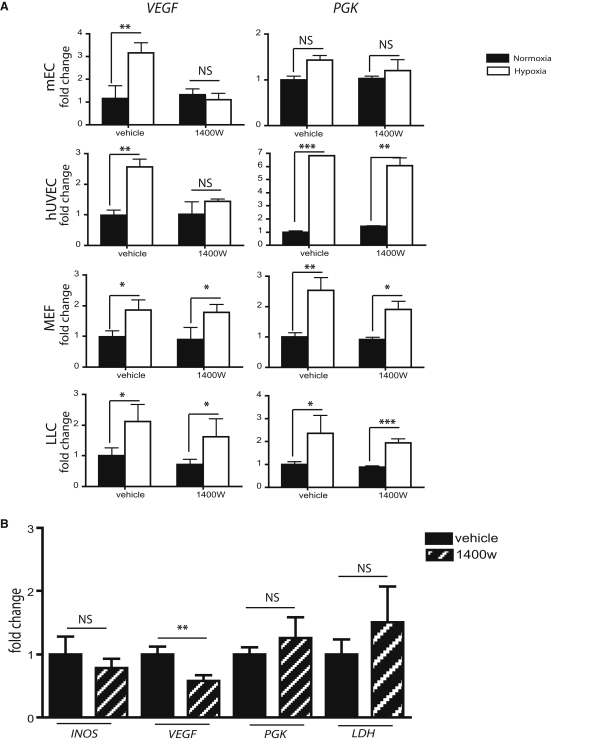
Inhibition of VEGF Expression upon iNOS Inhibition Is A Phenomenon Specific to Endothelial Cells (A) Quantitative PCR of wild-type mouse endothelial cells (mEC), human umbilical vascular endothelial cells (hUVEC), mouse embryonic fibroblasts (MEF), and Lewis lung cancer cells (LLC) exposed to normoxia or hypoxia (21% or 1% O_2_, respectively) in the presence or absence of 15 μM 1400W, to assess mRNA levels of VEGF and PGK (hypoxia and HIF-1α control). Data is average ± SEM of ratio of mRNA levels in sample compared to mRNA levels in WT untreated during normoxia. (B) Quantitative PCR of RNA extracted from subcutaneous LLC^GFP^ tumors in WT mice. Mice were treated with two intraperitoneal injections: the first, a 15 mg/kg 1400W (or respective saline control of equal volume) at 8 hr before sacrifice, and the second, a 10 mg/kg injection at 4 hr prior to sacrifice. An asterisk is placed above differences with a significance exceeding p < 0.05; ^∗∗^p < 0.01; ^∗∗∗^p < 0.001.

To determine the endothelial specificity of the effects described above, primary lung murine endothelial cells (mEC), human umbilical vein endothelial cells (hUVEC), murine embryonic fibroblasts (mEF), and Lewis lung carcinoma cells (LLC) were treated with the iNOS inhibitor 1400W (Figure 4A). Hypoxically-induced *VEGF* expression was suppressed in both endothelial cell cultures (mEC and hUVEC) treated with 1400W; however, there was no suppression of *VEGF* mRNA by 1400W in the fibroblast (mEF) or carcinoma (LLC) lines (Figure 4A). Another hypoxia-induced HIF target, the glycolytic enzyme phosphoglycerate kinase (*PGK*), is not as strongly affected by suppression of iNOS in any of the cell types examined (Figure 4A). This indicates that the suppression of *VEGF* expression through inhibition of iNOS may not simply affect *HIF-1*α induction, but rather act through a more complex mechanism.

### Inhibition of iNOS Reduces Tumor VEGF Expression In Vivo, but Does Not Significantly Affect Expression of Other HIF Target Genes

LLC xenograft tumors were assayed for net effects on gene expression when iNOS inhibitor was delivered at late stages of tumor growth. After 14 days of subcutaneous tumor expansion in wild-type animals, tumor-bearing mice received two injections with either a saline control or 1400W intraperitoneally. Four hours after the last injection, animals were sacrificed and tumors removed; the injection with 1400W caused a significant reduction in *VEGF* mRNA in the treated animals (Figure 4B). No statistically significant effect was seen in any of the other HIF targets assayed, including the two glycolytic enzymes lactate dehydrogenase (*LDH*) and *PGK*. This indicates that suppression of iNOS could be a useful modality for specifically suppressing VEGF expression in tumors, where it presumably acts at least in part on endothelial expression of the angiogenic factor.

### Differential Tumor Cell Migration through Endothelial Cells Is Both HIF Isoform- and NO-Dependent

**Figure 5:**
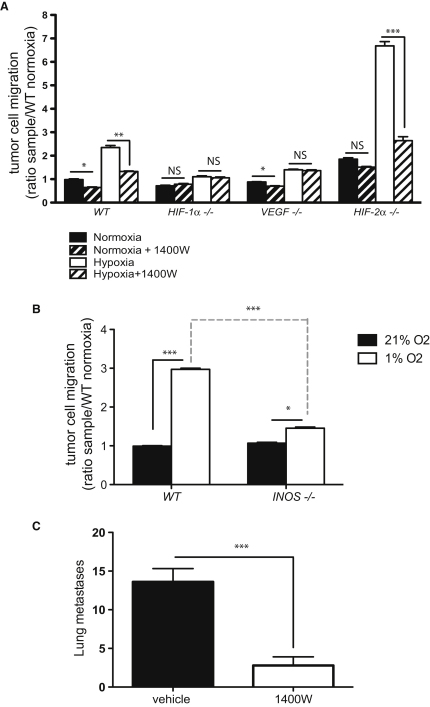
Hypoxia-Induced Tumor Cell Migration Is Inhibited in the Absence of Active iNOS (A) Endothelial monolayers of different genotypes were incubated with 15 μM of 1400W for 3 hr, and then washed to remove inhibitor. They were then incubated with LLC cells (in the absence of inhibitor) for 9 hr at either 21% or 1% O_2_, to assay for effects of inhibition of iNOS in endothelial cells on tumor cell migration. (B) Migration assay using *WT* and *iNOS null* EC monolayers. (C) Metastases in *WT* mice lungs 14 days posttail vein injection of 1 × 10^6^ LLC^GFP^ cells. Experimental mice received a total of two intraperitoneal injections of either control saline or 1400W, one at 8 hr, and one at 4 hr (the first of 15 mg/kg followed by 10 mg/kg) immediately prior to tumor cell injections. In this figure, data presented is average ± SEM. An asterisk is placed above differences with a significance exceeding p < 0.05; ^∗∗^p < 0.01; ^∗∗∗^p < 0.001.

To determine whether iNOS is the critical factor that modulates differential tumor cell migration through endothelial cells, tumor cell migration was scored in endothelial layers treated with either vehicle or 15 μM 1400W for 3 hr prior to tumor cell seeding. Inhibitor and vehicle were removed and endothelial cells were washed immediately prior to tumor cell introduction, in order to limit the effects of the inhibitor compound solely to the endothelial cells (Figure 5A). 1400W strongly suppressed hypoxia-induced tumor cell migration through *WT* endothelial cells; however, it had no suppressive effect on *HIF-1*α and *VEGF* null endothelial cells. Suppression with 1400W had the strongest effect on *HIF-2*α *null* endothelial cell layers, where the degree of suppression indicates that altered NO levels are likely a primary cause of elevated tumor cell migration in this genotype.

Migration assays performed using *iNOS null* EC indicates that, as expected, *iNOS* deletion results in reduced endothelial permeability to tumor cells (Figure 5B). A number of groups have shown that inhibition or deletion of inducible NOS results in suppression of tumor growth and metastasis (Ohtsu et al., 2010; Thomsen et al., 1997); we also found that treatment with 1400W prior to intravenous injection of 1 × 10^6^ LLC cells causes a significant reduction in metastatic success in vivo (Figure 5C).

### Differential Migration and NO Regulation Correlate with Metastatic Success in EC-Specific HIF Isoform Null Mice

As shown above, the loss of *HIF-1*α in endothelial cells reduces metastasis in mouse tumor models, and reduces tumor cell migration through an endothelial layer under hypoxia. The loss of *HIF-2*α in endothelium accelerates tumor cell migration in an NO-dependent manner. It was thus important to determine whether these observations on the role of the different isoforms in cultured endothelial cells is correlated with metastatic success in vivo.

**Figure 6:**
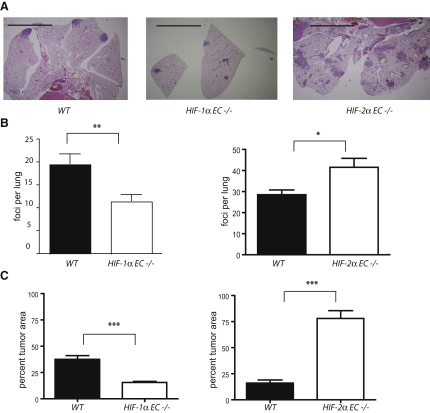
Endothelium-Specific Deletions of HIF-1α and HIF-2α Have Opposing Effects on Tumor Metastases LLC^GFP^ cells (1 × 10^6^) were injected via tail vein into HIF-1α^df^/Tie2Cre or *HIF-2*α*^df^/Tie2Cre* mice. All Cre-expressing and *WT* mice were lethally irradiated, and then received *WT* bone marrow, in order to ensure endothelial-exclusive deletion of gene of interest. Lungs were collected 14 days postinjection, fixed, and sectioned for hematoxylin and eosin staining. (A) Representative images of mouse lungs 14 days post-LLC^GFP^ tail vein injection. Scale bars represent 5 mm. (B) Number of metastases foci found in lungs of *HIF-1*α *null*, *HIF-2*α *null*, and their respective *WT* (i.e., df = double floxed/cre negative) controls. (C) Average of relative tumor area in lung sections obtained by quantifying tumor area versus whole lung area in every third section of whole lungs. In this figure, data presented is average ± SEM. An asterisk is placed above differences with a significance exceeding p < 0.05; ^∗∗^p < 0.01; ^∗∗∗^p < 0.001.

To model effects of this mutation on seeding of cells into the pulmonary endothelium and tumor cell extravasation, seeding of tumor cells into the tail vein in *WT*, *HIF-1*α EC null animals, and *HIF-2*α EC null animals was carried out. The mutant mice were created by *WT* bone marrow transplant into Tie2cre/conditional allele animals, as described above. Loss of endothelial cell *HIF-1*α significantly decreases metastatic success in this assay (Figure 6A). However, loss of *HIF-2*α in endothelial cells significantly increases both the number (Figure 6B) and size (Figure 6C) of pulmonary tumors in this experimental system.

## Discussion

The relationship of hypoxia to tumor progression has been well documented; hypoxia and hypoxia-associated necrosis have also been repeatedly linked to metastasis (De Jaeger et al., 1998). It is likely that hypoxic response is an intrinsic part of almost every stage of metastasis, through both hypoxia-induced tumor cell motility and increased vascular/endothelial permeability (Erler and Giaccia, 2006; Rofstad and Danielsen, 1999; Weis et al., 2004).

Clearly, a key to movement of tumor cells during hematogenous metastasis is transposition across endothelial barriers; the question being the degree to which this is influenced by a hypoxic response in the blood vessel itself. The hypoxic response in the endothelium is controlled to a significant extent by two transcription factors, HIF-1α and HIF-2α (Liu et al., 2011; Skuli and Simon, 2009; Tang et al., 2004; Ten and Pinsky, 2002). These in turn control a number of EC effectors: receptors and signal transduction molecules that shape endothelial and vascular behavior. One of the most potent and wide-ranging of these is NO. In the work described here, it is clear that NO levels are modified in EC by hypoxia in a manner that is controlled differentially by the HIF isoforms. Surprisingly, we also show that this control in turn correlates with either suppression or enhancement of metastasis.

A number of studies have shown that HIF-1α is a powerful regulator of the *NOS2*, or *iNOS* gene (Melillo et al., 1997; Ortiz-Masiá et al., 2010; Tafani et al., 2010; Zhou et al., 2009). Hypoxia acts in many cell types to induce iNOS, and thus to increase NO levels via the action of the synthase. It was recently shown that macrophages regulate NO homeostasis in part through Th1 cytokine stimulation and induction of *HIF-1*α transcriptionally; this in turn activates iNOS expression and ultimately increases NO production (Doedens et al., 2010). It has also been recently shown that HIF-2α induces *ARG1* expression following Th2 cytokine stimulation in macrophages (Takeda et al., 2010). The combined actions of the two HIF-α factors can thus regulate NO homeostasis (Takeda et al., 2010). The present study demonstrates that this differential and antagonistic action of the two HIF-α factors is not limited to cytokine-induced responses in macrophages: loss of *HIF-1*α limits *iNOS* expression in EC, and thus restricts NO production, and loss of *HIF-2*α results in lower levels of *ARG1*, and increased NO production.

NO levels have well-documented effects on EC function. Substantial literature exists describing the roles of NO in EC, both from extrinsic sources and intrinsic ones (Fukumura et al., 2006; Mark et al., 2004; Ziche and Morbidelli, 2009). However, endothelial-derived NO was previously thought to be primarily due to NO production through the eNOS enzyme (Fukumura et al., 2001); it is clear from the results described here that iNOS is a key source of NO in a hypoxic EC.

We also show here that iNOS itself is a key regulator of *VEGF-A* expression in endothelium, and that in its absence, fundamental aspects of VEGF-A function are disturbed, including VEGFR2 activation and tube formation. Although a number of workers have shown that iNOS is important for metastasis and can be linked to *VEGF-A* expression (Davie et al., 2007; Hosogi et al., 2005; Van der Wall and Palmer, 2006), the demonstration that it has an endothelial-specific role in those processes represents an intriguing avenue for tissue-specific VEGF-A inhibition.

One recent study showed that NO could act as an inhibitor of the prolyl hydroxylases, which in turn would act to increase HIF isoform stability and expression, and that this can act by interceding in the feedback loop where HIF isoforms themselves induce the expression of prolyl hydroxylases (Berchner-Pfannschmidt et al., 2007). Loss of *iNOS* would thus lead to increased degradation of HIF isoforms via increased activity of the prolyl hydroxylases; current work is underway to determine the role of this feedback loop in EC hypoxic response.

It is shown here that in EC, there are specific and opposing roles for the two HIF isoforms in regulating NO. Previously published studies of *HIF-2*α loss in EC either did not examine metastasis (Yamashita et al., 2008), or only scored animals qualitatively as metastatic or not after xenografts, i.e., did not count metastases or overall metastatic tumor burden (Skuli et al., 2009). It is clear that quantitative metastatic success is differentially influenced by the two HIF isoforms in ECs.

Interestingly, deletion of prolyl hydroxylase activity has profound effects on overall vessel maturation and pericyte coverage, in turn affecting vessel permeability and function (Coulon et al., 2010; Mazzone et al., 2009; Schneider et al., 2010). As decreased or altered hydroxylation will affect availability and activity of both HIF isoforms, understanding the relationships between individual hydroxylases and individual HIF isoforms will be an essential parameter to completely understand, in order to ultimately predict the effect of pharmacological hydroxylase inhibitors on tumor progression and metastasis.

**Figure 7:**
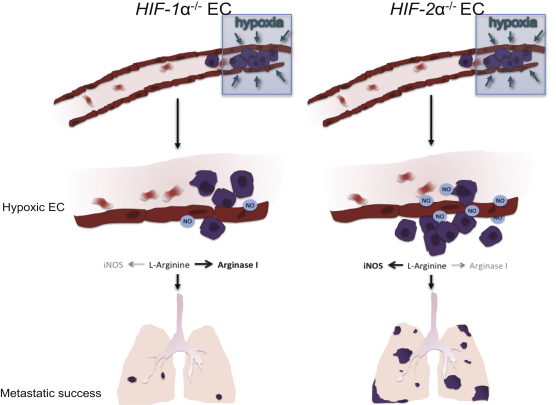
Differential Functions of HIF-1 and HIF-2 during Metastasis Depend on Differential NO Synthesis As shown, HIF-1 and HIF-2 response in endothelial cells gives rise to differing levels of NO production following hypoxic induction of the factors: in this case, through hypoxic stress caused by tumor cell-induced blockage of a capillary. This, in turn, results in differing levels of metastatic success when one or the other HIF is genetically deleted from the endothelial cell. A remaining and important question is how hypoxic stress can cause differing levels of induction of the two HIF factors.

It should also be noted that we have demonstrated an important difference in the roles of HIF isoforms in regulating tumor progression, as shown in the model in Figure 7. The model indicates the differential effects of the HIF isoforms in endothelial cells: here, in a thrombotic focus of tumor cells clogging a tissue capillary. This vascular blockage would immediately cause localized drops in oxygen tension in both the tumor cells and surrounding stroma, including the local endothelial cells. Depending on how the thrombus resolves, the HIF isoforms in the endothelium could act to regulate NO homeostasis locally, and in turn affect metastatic success.

The homeostatic regulation of NO clearly depends on differential expression of HIF-1 and HIF-2; this expression may be regulated by temporal differences in the isoforms' response to hypoxia (Holmquist-Mengelbier et al., 2006), and/or by differing cytokine/growth factor responsiveness (Takeda et al., 2010) as well as other factors in the tumorigenic milieu. This model reinforces the still developing concept that these two HIF isoforms can act antagonistically, and not redundantly, to regulate biological processes in malignancy (Bertout et al., 2009; Gordan et al., 2007; Roberts et al., 2009).

A number of studies have argued that in cancer, particularly in the context of VHL deletion, HIF-2α can act as the driver for malignant cell progression and tumor angiogenesis (Haase, 2006; Kondo et al., 2002; Smith et al., 2005). The data presented here indicate that the tumor stroma and malignant cells likely employ HIF isoforms differently, and as pharmacological manipulation of HIF factors is evaluated, their impact on HIF isoforms at a tissue-specific level should thus be considered and evaluated.

## Experimental Procedures

### Animal Experiments

All animal studies were carried out according to the animal protocol approved by the UCSD Institutional Animal Care and Use Committee. Mice were maintained on a standard chow in a pathogen-free animal facility with 12 hr light and dark cycles.

Deletion of *HIF-1*α or *HIF-2*α in EC and myeloid cells was obtained by crossing *HIF-1*α*^df^* or *HIF-2*α*^df^* females (generated as previously described [Ryan et al., 1998 and Gruber et al., 2007, respectively]) to *HIF-1*α*^df^/Tie2Cre+* or *HIF-2*α*^df^/Tie2Cre+* males. Cre+ and Cre− females between 3 and 4 weeks of age were lethally irradiated (1,000 Rad) and received wild-type bone marrow (1 × 10^6^ bone marrow [BM] cells per mouse) to restore genotype of hematopoietic cells and generate endothelial cell null mice and comparable df/wild-type controls.

Tie2-specific deletion of HIF-1α in polyoma middle T (*PyMT*) tumor model was obtained by crossing *HIF-1*α*^df^* females with *HIF-1*α*^df^/Tie2-Cre+/PyMT+* males. Only virgin females were used in this study. After weaning at 3 weeks of age, mice were palpated once per week. Tumor onset age was when a 2 × 2 mm palpable solid mass appeared. Tumors were measured in two dimensions using digital calipers. Mice were sacrificed when any tumor had reached 1 × 1 cm (endpoint).

C57Bl/6 *WT* and *iNOS*-deficient mice in the C57Bl/6 background were purchased from the Jackson Laboratory (Bar Harbor, ME) and used to purify endothelial cells lacking iNOS.

### Histology and Immunohistochemistry

Lung and tumor tissues were fixed in 10% phosphate-buffered formalin (Fisher Scientific) for 16 hr and embedded in paraffin. Lung metastases were counted in hematoxylin and eosin (H&E) stained sections as previously described (Liao et al., 2007). Blood vessels in tumors were detected using rat anti-mouse CD34 antibody (1:100 dilution, NB600-1071, Novus). Secondary antibody for CD34 staining is biotinylated goat anti-rat IgG (1:200 dilution, sc-2041, Santa Cruz Biotechnology). Staining was revealed using Vectastain ABC kit (Vector Laboratories). Microvessel density was quantified using a Chalkley graticule eyepiece.

### Generation of LLC^GFP^ Cells

Lewis lung carcinoma cells were infected with a lentivirus expressing GFP (made available by Dr. Cornelis Murre, University of California, San Diego) according to the standard protocol for retrovirus infection. Single clones from FACS-sorted GFP-positive cells were selected, and the clone used in this showing the highest GFP expression was used. LLC^GFP^ cells were cultured in DMEM (11965-092, Invitrogen) supplemented with 10% fetal bovine serum (26140-079, Invitrogen), 100 U ml^−1^ penicillin, and 100μg ml^−1^ streptomycin.

### Tumor Cell Extravasation

A total of 1 × 10^6^ LLC^GFP^ cells in 200 μl sterile PBS were injected intravenously into bone marrow-reconstituted *WT* and *HIF-1*α and *HIF-2*α *EC null* mice. Lungs were excised 14 days postinjection and fixed in 10% formalin for histological analysis. The number of tumors were counted in H&E stained 10 μm serial lung sections of the whole lungs and compared between *WT* and *EC null* mice. The area of each lung tumor of every third section was measured and compared to the whole lung area in the section for an assessment of tumor burden, using ImageJ software.

### Tumor Cell Intravasation

Right mammary gland pad 4 was surgically cleared in 3–4-week-old *HIF-1*α*^df^* and *HIF-1*α*^df^/Tie2-Cre+* female mice. Removal of ductal tree was confirmed by carmine whole-mount staining. Two months after surgery, bone marrow reconstitution was performed on these mice to obtain *WT* and *EC null* mice with cleared fat pads. Six weeks post-BM transplantation, 2.5 × 10^6^ LLC^GFP^ cells in 50 μl sterile PBS were implanted into the cleared mammary gland. Mice were sacrificed 21 days later, and tumors were weighed, lungs removed and fixed for histological analysis of metastasis, and blood was collected by cardiac puncture. Relative abundance of genomic GFP in blood was determined by quantitative PCR to assess the presence of circulating tumor cells.

### Quantitative Real-Time PCR

Genomic DNA was isolated using DNeasy Blood and Tissue Kit (QIAGEN). *GFP* DNA was quantified using an ABI Prism 7700 Sequence Detector (Applied Biosystems) and normalized to *VEGF* DNA levels. Conditions for the PCR: one 10-min incubation at 95°C, followed by 40 cycles of 15 s at 95°C and 1 min at 60°C. Primers used in this study include:

GFP forward, 5′-GGAGCGCACCATCTTCTTCA-3′;
GFP reverse, 5′-AGGGTGTCGCCCTCGAA-3′;
VEGF forward, 5′-CTATGGAGGCCAGAAGAGGGTAT-3′;
VEGF reverse, 5′-CCCACATCAGGTGGCTCATAA-3′; and
VEGF probe, 5′-(6FAM)AGATCCCTTGAAGCTAG(MGBNFQ)-3′.

For quantitative analysis of steady-state mRNA levels, total RNA was extracted from normoxia- and hypoxia-treated cells, using UltraClean Tissue and Cells RNA Isolation Kit (MoBio). cDNA was synthesized from 1 μg of total RNA, using Superscript III (Invitrogen) according to the manufacturer's instructions. Relative abundance of transcripts of interest was assessed by Q-PCR following normalization to β*-ACTIN* transcript levels. Primer pair and primer/probe sets used were as follows:

mb-actin forward, 5′-AGGCCCAGAGCAAGAGAGG-3′;
mb-actin reverse, 5′-TACATGGCTGGGGTGTTGAA-3′.
mVEGF total forward, 5′-ATCCGCATGATCTGCATGG-3′;
mVEGF total reverse, 5′-AGTCCCATGAAGTGATCAAGTTCA-3′;
mVEGF probe, 5′-[6∼FAM]-TGCCCACGTCAGAGAGCAACATCAC-[BHQ1a-Q]-3′.
mPGK forward, 5′-CAAATTTGATGAGAATGCCAAGACT-3′;
mPGK reverse, 5′-TTCTTGCTGCTCTCAGTACCACA-3′;
mPGK probe, 5′-[6∼FAM]-TATACCTGCTGGCTGGATGGGCTTGGACT-[BHQ1a-Q]-3′;
mArginase forward, 5′- AACACGGCAGTGGCTTTAACC-3′ (Takeda et al., 2010);
mArginase reverse, 5′ GGTTTTCATGTGGCGCATTC-3′ (Takeda et al., 2010);
miNOS: TaqMan Gene Expression Assay Mm00440488_m1 (Applied Biosystems); and
hiNOS: TaqMan Gene Expression Assay Hs01075521_m1 (Applied Biosystems).

Data are presented as average ± SEM of fold-change (ratio) between each sample and the WT normoxic control.

### Isolation of Primary Endothelial Cells

Primary endothelial cells were isolated and cultured from lungs of *HIF-1*α*^df^*, *HIF-2*α*^df^*, *VEGF^df^*, or *iNOS null* mice, as previously described (Dong et al., 1997; Tang et al., 2004) with the following modifications: the lungs were excised, minced, and digested for 90 min at 37°C in 2 mg/ml collagenase type I (Roche) in HBSS containing 2 mM CaCl_2_, 2 mM MgSO_4_, and 20 mM HEPES. The digest was filtered through a 70 μm nylon cell strainer and washed once in HBSS. Pellet was then resuspended in PBS containing 0.1% BSA and incubated with anti-CD31-coated magnetic beads (Dynal, Invitrogen) for 1 hr at 4°C. Cells and beads were plated in endothelial cell growth medium (ECGM) consisting of low glucose DMEM:F12 with 1% penicillin/streptomycin, 1% nonessential aminoacids, 2 mM sodium pyruvate, buffered with 20 mM HEPES and containing 20% FBS (Omega Scientific, Tarzana, CA), 20 μg/ml Heparin (Sigma, St. Louis, MO), and 75 μg/ml endothelial mitogens (Biomedical Technologies). Cell identity and culture purity were confirmed by immunodetection with anti-VE-cadherin (Santa Cruz Biotechnology, sc-6458), and LDL uptake.

### Transendothelial Cell Migration Assay

After 12–14 days, cells from double-floxed genotypes, in the original isolates (p0) were infected with adenovirus expressing Cre recombinase (for *HIF-1*α, *HIF-2*α, or *VEGF* deletion) or adenovirus expressing β-gal, for control *WT* cells. All migration experiments shown were performed using endothelial cell monolayers generated from P1 or P2 cells. Endothelial cells (5 × 10^5^) were seeded into COSTAR transwells (6.5 mm diameter, pore size 8 μm; Corning, NY) grown until confluent. Migration of tumor cells was examined by seeding 5 × 10^5^ LLC^GFP^ cells onto the endothelial monolayer and the inserts incubated for 9 hr under 21% (kept in normoxic growth incubator) or 1% oxygen (transferred to hypoxic incubator), with media on both upper and lower chambers. Inserts were stained with 0.1% crystal violet in 10% ethanol and mounted onto glass slides. Migrated cells were counted in five random fields per insert at 100× magnification. Average was calculated from a minimum of three inserts per treatment. Data is expressed as average ± SEM of the ratio between migrated cells observed through each endothelial genotype and/or treatment and the migration observed through the WT endothelium under normoxic conditions.

Effect of iNOS inhibition in endothelial cells on tumor cell migration was done by preincubation of endothelial monolayer with 15 μM of 1400W for 3 hr prior to the migration experiment (normoxia and hypoxia for 9 hr), which occurred in the absence of the inhibitor.

### Immunoprecipitation of VEGFR2

Subconfluent (∼80%) EC cultures in 6-well plate wells were incubated at 21% (normoxia) or 1% (hypoxia) for 9 hr, rinsed briefly with cold PBS, and immediately frozen in liquid N_2_. Cells were lysed in RIPA buffer (Cell Signaling, 9806S) containing protease inhibitors (Minitab, Roche), and 100 mM PMSF. Protein was quantified in clarified lysates and equal amounts were used for pull down of VEGFR2 (Santa Cruz Biotechnologies, sc-504). Whole cell lysate and primary antibody were preincubated O/N in a final volume of 750 μL, at 4°C. Protein A/G Plus Agarose beads (Santa Cruz Biotechnologies, sc-2003) were added and incubated O/N with end-over-end tumbling at 4°C. A/G beads were washed and bound protein resolved by SDS-polyacrylamide gel electrophoresis, and transferred to PVDF membrane for immunoblotting with anti-VEGFR2 (sc-504) and antiphosphotyrosine antibody (4G10 Platinum, Millipore) to detect and quantify the activated form of VEGFR2.

### Immunoblotting

Equal amounts of protein (15 μg) for normoxia- and hypoxia-treated cells were loaded onto 3%–8% acrylamide Tris-Glycine gels (Invitrogen) and transferred to PVDF membrane (Millipore), according to established western blotting procedures. Primary antibodies were used at 1:1,000 dilutions unless otherwise stated (iNOS [sc-651], eNOS [BD 610296], HIF-1α [Novus Biologicals NB-100-049], HIF-2α [R&D AF2997]). Proteins of interest were detected following secondary incubation with HRP-conjugated antibodies and ECL Plus chemiluminescence detection kit (Amersham).

### NO Measurements

Endothelial cells were grown to confluence and incubated for 48 hr at 21% or 0.5% O_2_ in growth media. Nitrate and nitrite levels were quantified in conditioned media using Sievers Nitric Oxide Analyzer (NOA 280i) according to the manufacturer's instructions. EC production of NO during normoxia and hypoxia was quantified by measuring the nitrites in the growth medium, which is likely an underestimation of the NO produced. Because the EC growth medium is saturated with nitrates, the contribution of EC production of NO to this pool was undetectable.

### Statistical Analyses

Statistical analysis in all cases (Q-PCR, NO levels, migration assays) was done using unpaired Student's t test, where values observed for each sample were routinely compared to the ones found in wild-type controls under normoxic/untreated conditions. Data are expressed as mean ± SEM unless otherwise stated.
